# Supplementary material for: Interpretable Machine Learning of Nanoparticle Stability through Topological Layer Embeddings
Source: J Phys Chem A. 2026 Jun 27;130(27):5293–307. doi: 10.1021/acs.jpca.6c01508 (PMC13359354; doi:10.1021/acs.jpca.6c01508)
Supplement: Supplementary file 1 [file jp6c01508_si_001.pdf]

Supporting Information for Interpretable Machine Learning of Nanoparticle Stability through Topological Layer Embeddings

Felipe Hawthorne,[†,‡] Leandro Seixas,[¶] James M. Almeida,[§] Cristiano F. Woellner,[*,†,‡] and Raphael M. Tromer[*,‖]

†*Department of Physics, Federal University of Paraná, 81530-015, Curitiba, Paraná, Brazil*

‡*Interdisciplinary Center for Science, Technology, and Innovation (CICTI), Federal University of Paraná, 81530-000, Curitiba, Paraná, Brazil*

¶*Instituto de Física Teórica, Universidade Estadual Paulista, 01140-070, São Paulo, São Paulo, Brazil*

§*Ilum School of Science, Brazilian Center for Research in Energy and Materials (CNPEM), 13083-970, Campinas, São Paulo, Brazil.*

‖*University of Brasília, Institute of Physics, 70910-900, Brasília, Federal District, Brazil.*

E-mail: woellner@ufpr.br; raphael.tromer@unb.br

Supplementary figures

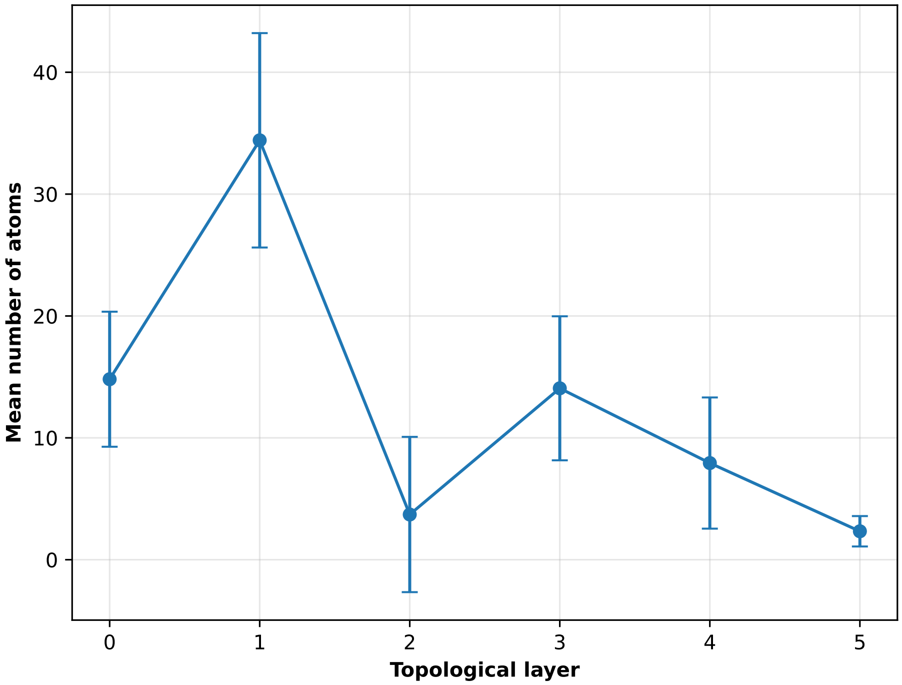

Figure S1: Mean atoms per layer.

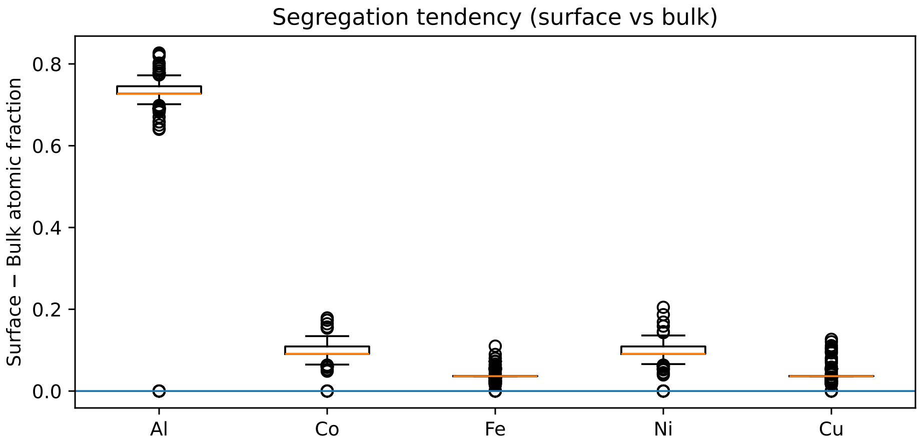

Figure S2: Surface–bulk segregation tendencies expressed as the difference between surface and bulk atomic fractions. Al exhibits a strong and systematic enrichment at the surface, whereas transition metal species show substantially weaker segregation. The consistency of these trends across the dataset highlights the robustness of layer-dependent chemical heterogeneity and confirms that the descriptor encodes physically meaningful surface–core contrasts beyond global stoichiometry.

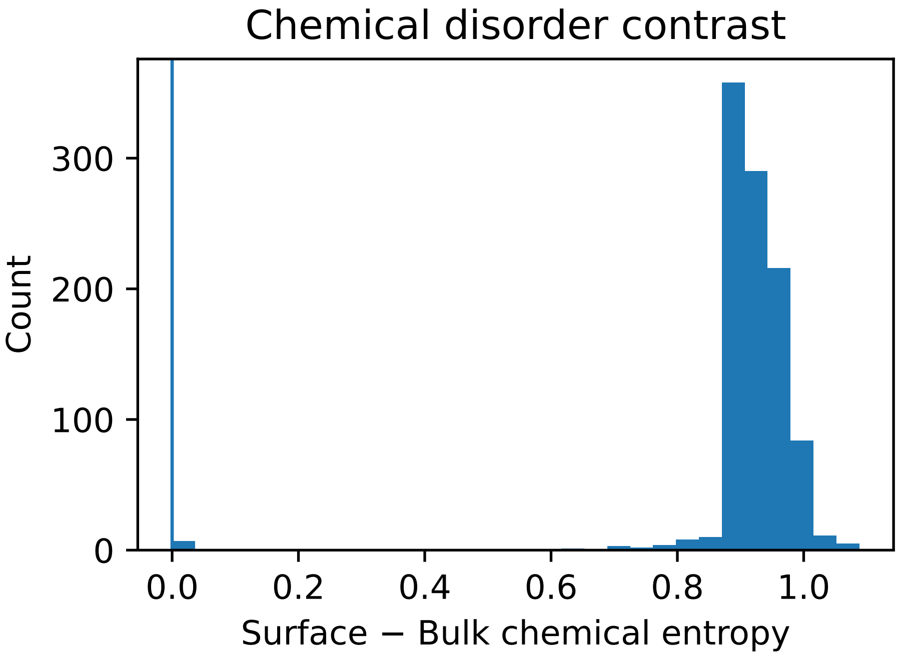

Figure S3: Distribution of the chemical entropy contrast between surface and bulk regions, defined as $\Delta S_{\mathrm{chem}} = S_{\mathrm{chem}}^{\mathrm{surface}} - S_{\mathrm{chem}}^{\mathrm{bulk}}$, across the full nanoparticle dataset. Positive values indicate enhanced chemical disorder at the surface relative to the interior. The asymmetric distribution, centered at positive ΔS_{chem}, demonstrates that surface regions systematically accommodate a higher degree of chemical mixing, reflecting the combined effects of reduced coordination, segregation-driven compositional fluctuations, and relaxed bonding constraints. This result provides quantitative evidence that chemically complex nanoparticles exhibit intrinsically heterogeneous disorder landscapes, which cannot be captured by global descriptors alone and are naturally resolved by the proposed layer-resolved representation.

Hyperparameter optimization and cross-validation

All XGBoost models were trained on an 80/20 train–test split with a fixed random seed (42). Hyperparameters were optimized with the Tree-structured Parzen Estimator (TPE) sampler in Optuna; the search space and resulting best values are summarized in Table S1. For each Optuna trial, the objective was the mean absolute error (MAE) averaged over a 5-fold cross-validation on the training partition, so the test set was never seen during model selection. The number of Optuna trials was set to 300 for the ranking-task model and to 60 for the layer-weighting study, both empirically chosen to ensure convergence of the best-trial MAE.

Table S1: XGBoost hyperparameter search ranges and resulting best values for the ranking-task model and the layer-weighting study.

Hyperparameter	Search range (rank)	Search range (layer-weighting)
`n_estimators`	$[200, 800]$	$[300, 1200]$
`max_depth`	$[3, 8]$	$[3, 7]$
`learning_rate`	$[10^{-3}, 0.2]$ (log)	$[10^{-2}, 0.1]$
`subsample`	$[0.7, 1.0]$	$[0.6, 1.0]$
`colsample_bytree`	$[0.7, 1.0]$	$[0.6, 1.0]$
`reg_lambda`	–	$[0.1, 5.0]$
Optuna trials	300	60
CV folds (inner)	5	5

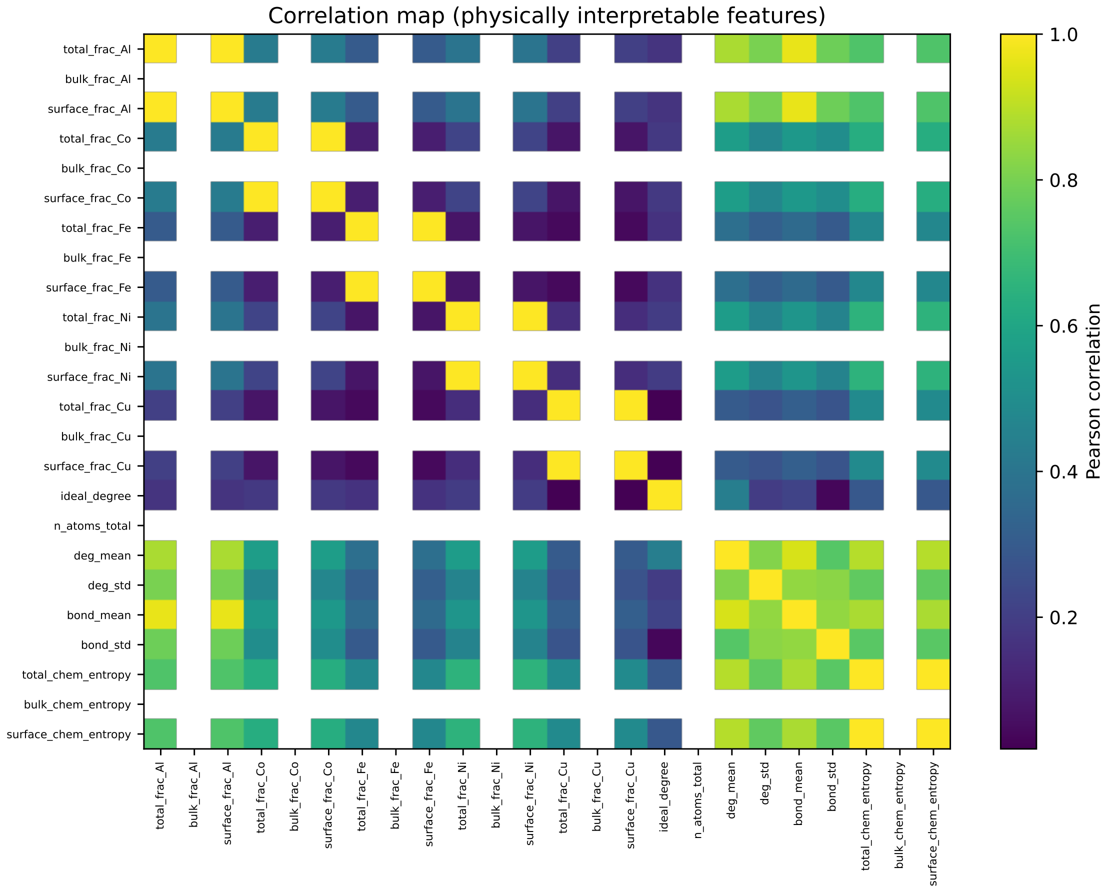

Figure S4: Correlation map between selected physically interpretable descriptors, including global, bulk, and surface-resolved composition, coordination, bonding, and chemical disorder metrics. Strong correlations are observed among chemically related quantities (e.g., total, bulk, and surface fractions of the same element), reflecting the constrained global stoichiometry, while cross-correlations between compositional, topological, and disorder-related features remain moderate. Notably, chemical entropy descriptors exhibit systematic correlations with coordination and bond-length statistics, highlighting the coupled nature of chemical disorder and local topology in chemically complex nanoparticles. The absence of excessive collinearity across distinct descriptor families demonstrates that the proposed layer-resolved representation encodes complementary and physically meaningful information across surface and bulk environments.
